# A mixed-method study examined the reasons why pregnant women late initiate antenatal care in Northeast Ethiopia

**DOI:** 10.1371/journal.pone.0288922

**Published:** 2023-07-24

**Authors:** Melaku Shewaye, Niguss Cherie, Asressie Molla, Abebe Tsegaw, Chalachew Yenew, Dessalegn Tamiru, Sefineh Fenta Feleke, Anteneh Mengist Dessie

**Affiliations:** 1 Department of Public Health, College of Medicine and Health Sciences, Wollo University, Dessie, Ethiopia; 2 Department of Public Health, College of Health Sciences, Debre Tabor University, Debre Tabor, Ethiopia; 3 Department of Public Health, College of Medicine and Health Sciences, Jimma University, Jimma, Ethiopia; 4 Department of Public Health, College of Medicine and Health Science, Woldia University, Woldia, Ethiopia; Arba Minch University, ETHIOPIA

## Abstract

**Background:**

Timely initiation of antenatal care visits is crucial for ensuring optimal care and health outcomes for women and children. However, late antenatal care initiation among pregnant women in low-income countries including Ethiopia is acknowledged in the literature. Therefore, this study examined why pregnant women initiate antenatal care late in Northeast Ethiopia.

**Methods:**

This study was done using a mixed design approach that included phenomenology and retrospective cross-sectional designs. A retrospective cross-sectional study was deployed among randomly selected 632 medical charts of women who had antenatal care visit in Legambo District primary hospital and health centers. Kaplan Meier curve was used to estimate survival time. Bi-variable and multivariable Cox-proportional hazard regression models were fitted using R-studio-1.2.5033 to identify independent predictors of antenatal care initiation time. Six vignette-based focused group discussions were held with pregnant women who had been purposefully selected. Then, a qualitative content analysis that was informed by the phenomenological theory was done.

**Results:**

This study indicated that only 195 (30.9%) of women timely initiated their antenatal care follow-up. In a survival analysis, all 632 women contributed 12,474 person-weeks giving a timely antenatal care initiation rate of 15.6 per 1000 person-weeks. According to the multivariable Cox regression models, late antenatal are initiation was found among pregnant women with an unknown last normal menstruation period, no living children, no bad obstetric history, not accompanied by their partner during antenatal care visit, and who lived in a >5-kilometer radius of health facility. In a qualitative analysis, being uncertain whether the pregnancy had occurred, believing that they have a very low probability of experiencing any complications, negative experience with healthcare providers, negative influence from their immediate contacts such as the mother and mother-in-law, and socio-cultural beliefs like "Qare" were found as major reasons why they started antenatal care follow-up late.

**Conclusion:**

Most pregnant women in Legambo district, northeastern Ethiopia, initiate their antenatal care visits late. Based on the findings of this study, strengthening the pregnant women identification program and performing regular pregnant women’s conference will help to achieve early initiation of antenatal care visits. To lessen the negative influence of their immediate contacts, it will also good to include these old moms and husbands in the conference of pregnant women.

## Introduction

Maternal mortality from preventable pregnancy-related complications remains exceptionally high, with 300,000 maternal deaths globally, reported in 2015 [1]. Around 99% of maternal deaths worldwide during the reporting period occurred in low-income countries (LICs), with 66% of these deaths occurring in sub-Saharan African countries (SSACs) [2]. The case of Ethiopia is not much different; the maternal mortality ratio (MMR) was 412 per 100,000 live births as of a 2016 Ethiopian Demographic Health Survey (EDHS) report [3]. However, the Sustainable Development Goal (SDG), which Ethiopia has also been committed to achieving, aims to reduce maternal mortality to 70 per 100,000 live births by 2030.

One of the main reasons for this high maternal mortality ratio around the world in this era was the late initiation of antenatal care (ANC) visits [4]. When a woman initiates and /or attends ANC follow-up late, very little can be done to prevent, early detect, appropriately manage, and timely refer women with complications. This leads to a higher risk of complications which could lead to morbidity, disability and mortality [5].

Even though the timely initiation of ANC visits is recognized as the cornerstone of maternity and newborn health care, most pregnant women attend their first ANC visit late. The late ANC initiation magnitude is different for countries. The magnitude of late antenatal care initiation was reported as 80% in Nigeria [6]. A study conducted in East African countries also indicated that late ANC initiation was 49.8%, 60.5%, and 61.0% in Tanzania, Kenya, and Democratic Republic of Congo, respectively [7]. According to the 2016 EDHS report, 80% of women had their first ANC visit after 16 weeks of gestational age [3]. Studies conducted in Ambo and Addis Zemen, Ethiopia showed that the late initiation of ANC was 86.8% and 52.5%, respectively [8, 9].

Studies conducted in Ethiopia revealed that several factors, including maternal age, marital status, residence, maternal education, employment status, economic status, health insurance, traveling time, parity, knowledge about ANC, history of abortion, previous birth outcome, experience of service utilization, pregnancy-related complications, want status of the pregnancy, birth preparedness plan, and pregnancy intention had an influence on the timing of ANC initiation [4, 8, 10].

Few studies on the timing of antenatal care have been conducted in Ethiopia, and none has focused on the Lagambo district. There is also very limited qualitative literature which tried to understand the reasons for late ANC initiation in Ethiopia [4]. Therefore, the main objective of the study was to examine the reasons why pregnant women late initiate antenatal care among pregnant women of Legambo District, Northeast Ethiopia using a mixed method approach.

## Materials and methods

### Study area and period

The study was carried out in Legambo District, South Wollo administrative zone of Amhara region, Ethiopia from 1 January 2020 to 30 June 2020. The district situated at an altitude of 1500 to 3700 meters above sea level, is 501 km far from Addis Ababa and 600km from Bahir Dar. The total population of the area has been estimated to be 197,199 of whom 99,362 (50.38%) are women. Among those 46,499 (23.57%) are estimated to be reproductive age grouped women. Pregnant women constituted around 3.37% of all reproductive age group women. As to the health system structure, the district has 1 primary Hospital, 9 Health centers, 40 Health posts, and 7 private clinics.

### Study design

A retrospective cross-sectional study design was utilized for the quantitative part, while a qualitative content analysis informed by phenomenological theory was taken into account for the qualitative section. Thus, the study employed a mixed-method approach.

### Eligibility criteria

For the quantitative part of this study, all women who registered on the ANC registration book in Legambo district primary hospital and health centers from 1 July 2017 to 30 December 2019 were included. Only the women’s medical charts that contain incomplete record about their ANC initiation time has been excluded from the study.

In order to have the qualitative data, all pregnant women and their husbands who were willing to give consent and give information about factors that affect the starting time of the ANC follow-up were included. Husbands were included in the Focused Group Discussion (FGD) because of the following reasons. First, women will come free to the FGD session if they are with their husbands. Second, husbands are also likely to have insight into the research questions. Furthermore, in the surround of Wollo including our study area, women are free to talk under the present of their husband. Therefore, the presence of husbands may have no effect on our conversation; rather, it will be for the better. Individuals who cannot fully attend the FGD session due to illness, lack of time, or other reasons were not included in the this discussion.

### Sample size determination

Quantitative part: for this part of the study, sample size was calculated using the Epi Info 7 sample size calculation for cohort or cross-sectional design by considering the parity (nulliparous and multiparous) as a significant predictor of ANC initiation time from previous national studies [11]. The assumptions were power = 90%, confidence interval = 95%, Ratio (number in exposed: unexposed) = 1:1. The maximum estimated sample size was 632.

For the qualitative section, six vignette based FGDs were conducted with pregnant women and their husbands by considering data redundancy as a cut point for information saturation. Six individuals (3 pregnant women with their husband) were participated in a single FGD. Hence, 36 individuals (18 pregnant women and 18 husband) were participated in the whole FGD.

### Sampling procedures

Quantitative part: all 10 public health facilities (1 primary hospital and 9 health centers) were included in the study. The number of women who had ANC visit in each health facility during the specified period (1 July 2017 to 30 December 2019) was obtained. Then, using the proportional allocation formula, the total sample was shared with all facilities. The women’s medical charts in each health facility were then selected using a simple random sampling technique after a sampling frame had been created by taking the women’s medical record numbers from the ANC registration book. Finally, all qualitative data were collected from the selected medical charts of women.

Qualitative part: a purposive sampling technique was used to strategically select those pregnant women and their husbands who had in-depth information about the subject matter of the study. Health extension workers referred us on to suitable pregnant women who will have in-depth information about the subject matter of the study.

### Variables of the study

The dependent variable of this study was time of ANC initiation. Whereas, demographic factors (maternal age, residence, marital status, number of children alive), obstetric factors (gravidity, parity, high-risk current pregnancy, recognition of pregnancy/ Last Normal Menstrual Period (LNMP)), past obstetric history (stillbirth, abortion, caesarian section, the abnormal weight of the newborn, hospital admission for the last pregnancy, mode of delivery, delivery outcome, time of first ANC visit during the previous pregnancy), chronic illness, participation in ANC conferences, performance status of the health developmental army, and socio-cultural and behavioral factors were the independent variables addressed in this study.

### Data collection procedure

Quantitative data were collected as follow. Following the selection of the women’s medical chart, the gestational age at which they receive their first ANC was first determined. Women who receive their first ANC before sixteen weeks of gestation are referred to as "timely starters," whereas those who do so at or after that point are considered "late comers" [12]. The gestational age was measured in weeks and was taken to be the survival time. All information relating to demographic, obstetric, gynecologic, and general medical characteristics were retrieved from the medical charts of women using an information extraction sheet. The data were collected by well-trained ten BSc nurses after having a two days of training, and supervised by two MSc holder reproductive health specialists.

In order to collect the qualitative data, Vignette based FGD were considered. The FGD participants were asked to discuss about all the barriers in the community to starting ANC visits early and things that will make them to start ANC late. A short story of a hypothetical woman about her ANC experience and the cultural belief inside her community was presented as a Vignette, and they were asked to comment on how they think the character in the story would feel or act in the given situation, or what they would do themselves. It shifted the focus away from the participants and allowed them to share personal experiences freely. Information about the reasons behind the late start of ANC follow-up was obtained in this way and all data was recorded using a digital voice recorder. In addition to audio recording, notes were taken. A senior & experienced journalist from the woreda government communication affairs office who not only assumed the ordinary position of a data collector, but rather a critical interpreter of verbal & emotional talks of the study subjects collected the data. Both the researcher and data collector were familiar with the social and cultural norms present in the district under study, and the researcher has personal experiences that may have informed the development of the vignettes and the FGD questions.

#### Trustworthiness of the study

The triangulation of different data sources and data collection approaches (qualitative and quantitative) was used as one strategy to maintain trustworthiness in this study. Purposive sampling was also used to maximize specific data relative to the context in which it was collected. Adequate details on the site, participants, and methods or procedures used to collect data have been given above that help other researchers evaluate whether the results are applicable to other situations. Rigor data processing has been also done to make the data should speak for itself.

### Data processing and analysis

#### Quantitative data processing and analysis

Data were entered using Epi—data version 3.1 for cleaning & exported to RStudio-1.2.5033 for analysis. Basic descriptive and summary statistics were engaged to demonstrate the distribution of study subjects concerning their baseline characteristics. The survival time was figured as the gestational age in weeks at the time of to first ANC consultation (time from pregnancy to the first ANC visit). The hazard ratio (HR) for each explanatory variable and the overall rate of ANC uptake at different times within the range of our observation was computed using a life table. Cox regression deals with time-to-event data and is interested to know how long it takes for something to happen and its predictors. In this type of regression, the outcome variable should have a binary response and the survival time (in our case, time from pregnancy to first ANC visit) recorded for each observation. Hence, Cox proportional hazard regression model was used to identify the predictors of ANC initiation time. Their significance was, also, determined in the independent bi-variable, Cox regression model before controlling for the variables and estimating the crude hazard ratio (CHR) in the multivariable Cox regression models.

Variables having *P*–values of < 0.25 were entered into the multivariable model and those with p-value < 0.05 were considered as the significant predictors of the time to first ANC initiation. Furthermore, a 95% confidence interval (CI) was built around each of the HRs to further establish their significance. Kaplan–Meier curve was used to estimate the survival time for first ANC initiation after experiencing the pregnancy. The proportional hazards assumption was checked using statistical tests based on the scaled Schoenfeld residuals. Accordingly, the global test showed that the proportional hazard assumption was satisfied for the model as a whole with p-value of 0.25 and the detail of it also confirmed that each variable in the model satisfied the proportional hazard assumption with a p-value of >0.05.

#### Qualitative data processing and analysis

The qualitative data generated from the Vignette based FGDs within the group of women who were pregnant during the study period and their husbands have followed the principles of thematic content analysis. After listening to audio records several times, transcription of every word of the participants was considered accompanied by labeling and archiving of the narration into a pile of labor arching file system. Thus, data was organized by questions to look across all respondents and their answers to identify consistencies and differences. Manual coding of the copied content of transcripts, then, was done to identify factors affecting the timing of ANC booking. Two Health education and promotion instructors from Meqedela University coded selected transcripts in the first instance and compared them for consistency.

Then, discrepancies were appreciated after the investigator appraised the coding and emerged category results, and the rest transcripts were divided among the coders, and coding was completed independently. Grouping of codes into mutually exclusive and exhaustive categories, as possible, to identify and built common themes across the dataset was also considered. To have a look over the relative importance of such categories, counting the frequencies the theme comes up with was taken into account.

Furthermore, the formation of super categories by combining some other categories was also considered to see how the parts relate to the converging single theme. Then, a short description was made for each category with quotes, as needed, from the text that illustrated the meaning. Eventually, cutting and sorting of the pre-labeled data into the corresponding pre-defined categories followed by attachment of meaning and significance was undertaken. Translation into English was done at a later stage after identifying meaningful themes.

### Ethics consideration

Ethical clearance was obtained from the Ethical Review Committee of Wollo University College of Medicine and Health Sciences School of Public Health. The head of health centers and the hospital chief executive officers were briefed on the objectives of the study, and permission to collect the data was obtained from them. Since the data were collected retrospectively by reviewing the chart and had no direct contact with the study participants informed consent was not presumably required here in the quantitative section. However, for the qualitative part, written informed consent was obtained from each participant after explaining the study’s goal, risks, benefits, and confidentiality. Whatsoever, this study was carried out in accordance with the principle of the declaration of Helsinki.

## Results

### Socio-demographic characteristics of the study population

Six hundred thirty two charts of pregnant women were involved for the quantitative analysis of this study. Two hundred ninety-two (46.2%) of these charts were in the 2018 year of study, while 220 (34.8%) and 120 (19%) were from the 2017 and 2019 study years, respectively. Four hundred thirty-five (68.8%) of the women are rural dwellers and the median age of the subjects was found to be 28 with an interquartile range of 26 to 31. Moreover, 199 (31.5%) of the pregnant women had accessed ANC-providing units out of a 5 Km radius (**Table 1**).

**Table 1:** Socio-demographic characteristics of the pregnant women in the study area, 2017–2019.

Variables	Frequency	Percentage
Age		
15–19	5	0.8
20–24	109	17.2
25–29	315	49.8
30–34	131	20.7
35–39	33	5.2
40–44	23	3.6
45–49	16	2.5
Marital status		
Singe	6	0.9
Married	615	96.8
Divorced	3	0.4
Widowed	8	1.2
Residence		
Rural	435	68.8
Urban	197	31.2
Number of children alive		
0	71	11.2
1–3	554	87.7
4–7	7	1.1
Commuting distance		
< 5 Km	433	68.5
≥5 Km	199	31.5

### Obstetric characteristics and ANC initiation

**Fig 1:**
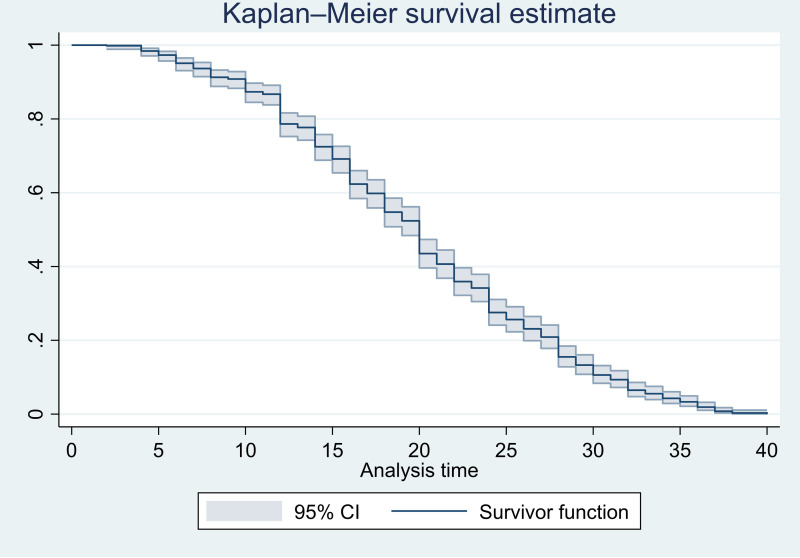
Kaplan-Meier survival estimate of time to first antenatal care visit among pregnant women in the study area, 2017–2019.

Concerning the obstetric history, 554 (87.7%) and 556 (88.0%) of pregnant women found multigravidas and multiparas, respectively. Furthermore, 115 (18.2%) of pregnant women had bad obstetric history credited, mostly, to abortion, stillbirth, and neonatal loss. Coming to the timing of ANC initiation by the study subjects 141 (22.3%), 345 (54.6%), and 146 (23.1%) received their first ANC at the first (0–13), second (14–26 weeks), and third trimesters (27–40 weeks) of the follow-up continuum. One hundred ninety-five (30.9%) of all 632 pregnant women initiated their ANC follow-up timely. In a survival analysis, all 632 women contributed 12,474 person-weeks giving a timely ANC initiation rate of 15.6 per 1000 person-weeks. The cumulative probability of 13, 26, and 32 weeks of survival (not started ANC visit) since pregnancy was 77.7%, 23.1%, and 6.5% respectively (**Fig 1**). Only 179 (28.3%) were accompanied by their partners during their ANC visit for the current pregnancy and 159 (25.2%) women did not know their LNMP.

### Determinants of the ANC initiation

According to the multivariable Cox regression models, women with unknown last normal menstrual period had 1.91 (AHR = 1.91, 95% CI = 1.59, 2.36) times higher hazard of late ANC initiation. Women who had no alive child and had 1–3 alive children were also 2.24 (AHR = 2.24, 95% CI = 1.76, 3.35) and 1.72 (AHR = 1.72, 95% CI = 1.78, 7.33) times at higher hazard of having late ANC initiation. The hazard of late ANC follow-up initiation among women who accompanied by partners during their ANC visits and can get ANC service within < 5 km radius of their residence was 0.56 (AHR = 0.56, 95% CI = 0.46, 0.68) and 0.62 (AHR = 0.62, 95% CI, 0.49, 0.71) times less likely than their counterpart. Similarly, those women who had bad obstetric history were 65% (AHR = 0.35, 95% CI = 0.279, 0.442) less hazard to initiate ANC follow-up late. In contrast to any other studies, women who have known chronic illness were 2.33 (AHR = 2.33, 95% CI = 1.56, 3.45) times higher hazard of having late ANC follow-up initiation (**Table 2**).

**Table 2:** Determinant factors of late ANC initiation of pregnant women in the study area.

Variables	Frequency	CHR	*P*-value	AHR	95% CI
LNMP					
Known	473	1		1	
Unknown	159	0.71	< 0.001	1.91	1.59–2.36
Number of children alive					
0	71	1.79	0.002	2.24	1.76–3.35
1–3	554	1.19	0.036	1.72	1.78–17.33
4–7	7	1		1	
Partner accompanied					
Yes	179	0.58	< 0.001	0.56	0.46–0.68
No	453	1		1	
Chronic illness					
Yes	30	1.54	0.003	2.33	1.56–3.45
No	602	1		1	
Bad obstetric history					
Yes	115	0.32	0.022	0.35	0.28–0.44
No	517	1		1	
Commuting distance					
< 5 Km	433	0.31	0.002	0.62	0.49–0.71
≥5 Km	199	1		1	

The qualitative approach also revealed several significant predictors that explore why women in the Legambo district initiate their antenatal care visit late. These stated reasons had been placed under five thematic areas: pregnancy uncertainty, belief that one is low-risk and misconception about ANC importance, influence of social networks, negative experience with healthcare providers, and harmful cultural beliefs and myths in the community.

#### Pregnancy uncertainty

Most vignette FGDs participants state that pregnancy uncertainty–being uncertain whether the pregnancy had occurred or not–was the major factors that made pregnant women to not have timely ANC initiation. The majority of the women who participated in the group talks assert this fact: that this uncertainty about whether the pregnancy had occurred or not was a result of a side effect of the previous contraceptives they used. A 26-year-old woman from FGD reported the following: *"…I had used contraceptive methods for two consecutive years*, *and my period had become irregular after I stopped it*. *When I went to a health facility in the meantime for other services*, *they announced to me that I was 24 weeks pregnant*. *I did not realize that I was pregnant until that time*. *My period was irregular*, *and I hadn’t experienced any pregnancy-related symptoms like nausea*, *vomiting*, *or weight gain*.*"*

#### Belief that one is low-risk and misconception about ANC importance

The Vignette based FGDs also reflected that the extent to which pregnant women view themselves as being at risk of bad pregnancy outcomes was poor. Some of the participants thought the absence of abnormal symptoms during pregnancy was a reflection of the normality of the fetus, inside. Hence, the need to early present for an ANC visit is not as necessary as if there is a complication. Along with this low risk perception, many of the participants did not appear to be aware of the benefits of ANC. As per the quote 32-years-old women from FDG: *"* … *All of my previous pregnancies*, *as well as this one*, *have gone smoothly*. *I will only visit a health facility if I experience a discomfort*. *Otherwise*, *what new things will the healthcare providers make for us if the pregnancy goes well*? *They simply examine us and tell us that if the pregnancy is going well*, *we can return home*. *There is no need*, *therefore*, *to go to the antenatal care visit if a woman is well*.*"*

#### Negative experience with the healthcare providers

Our study also established that poor handling given to pregnant women by healthcare providers during their previous ANC made them late or not come at all for ANC service if no complications were present. Participants reported that healthcare providers did not make pregnant women feel welcome during their ANC visit and this unfriendly care from healthcare providers led them to choose to stay at home if there were no complications. A 29-year-old woman said as follows: *"* … *I am actually telling you the truth*. *I felt discomfort during the whole period of my previous pregnancy and I came to the health facility frequently*. *However*, *the healthcare providers’ handling was not good*. *They even shouted at me*, *saying*, *"You are okay enough*, *why do you come frequently and make us busy*? *We are handling many pregnant women*. *You see*, *this type of poor handling in the health facility compels us to be late or not come at all for ANC service if there were no complications*.*"*

#### Influence of social networks

Alongside this, many participants repeatedly asserted that having different advice from other significant persons (i.e. mothers and mothers in law, husbands, neighbors, relatives, and friends) was one of the dynamics that made them uncertain to decide when to book for ANC services. The male participants in the discussion also indicated that social networks had an influence on their wives’ decisions regarding when to seek their first ANC service. Study participants were also asked what the role of Health Extension Workers (HEWs) and Health Development Army (HDA) leaders were while deciding when to make their first ANC visit. All participants ascertained that HEWs and HDA leaders had a substantial positive impact on the timing of their first ANC visit. A 21-year-old woman explained the negative and positive influence of social networks and health extension workers in their ANC visit initiation time: *"* … *In fact*, *my husband said to me that what you are going to do in a health facility if you are not sick since the pregnancy is too early*? *She is a health extension worker who referred me to the health facility*, *saying that you all should have antenatal care visits from the moment you find out you are pregnant*.*"*

#### Harmful cultural beliefs and myths

The socio-cultural beliefs and myths in the study district have played an important role in influencing the timing of ANC initiation. One of the traditional beliefs is called ‘Qare’, which claims that two pregnancies will antagonize each other if they get together on an occasion. Thus, if one of the pregnancies had something wrong, the effect will be reflected on to the other one. As per the participants, this effect may take the form of abortion, miss-timed labour, or abnormally behaved babies after delivery. For this reason, pregnant women are advised not to early reveal themselves in public gathering areas: one among which is the clinic where other pregnant women with different health problems are supposed to be there. They therefore preferred to visit health centers once their pregnancy had reached a mature stage, and they took traditional ‘shotella (having consecutive pregnancy loss)’ medication as a reserve protective mechanism. Findings in this regard were very consistent across all the groups. A 35-year-old woman described her experience of this issue as follows: " … *Obviously*, *if pregnant women present themselves in a public gathering place and a pregnant woman with a problem is there*, *the problem will be reflected in a woman with a healthy pregnancy*. *I know a woman who experienced this type of problem*. *She went to church during her early period of pregnancy and saw a mother with a problem of ‘shotella’ and the woman aborted her pregnancy*."

## Discussion

The main objective of the study was to estimate the ANC initiation and to identify corresponding determinants among pregnant women in Legambo District, South Wollo Zone, Amhara Region, Ethiopia. When we have a look over the median gestational age they initiate ANC visits was found to be 21.05 weeks (5.1 months), which is nearly comparable with study findings in Gamogofa, Arbaminch (5 months) [11], but slightly lower than from Nigeria’s (6 months survival) [13]. The difference may be due to study settings (population and geography) and Nigeria’s study used long-term Demography and Health Survey data in contrast to health management information system-based data. Further explorative analysis of this study showed that the cumulative percentage for uptake ANC services at 16 weeks of gestation was increased along with time pass 22% (2017/18), 26% (2018/19), and 49.2% (2019/20) with a pooled effect to be 30.9%. This finding is comparable with the study reports from Nigeria (32.3%) [13] and Tigray (32.7%) [14].

Several significant factors that explore why women in the Legambo district initiate their ANC visit late has been found from both the quantitative and qualitative approach of this study. Pregnant women who had no bad obstetric outcomes prior to the current pregnancy had a 2.9 times higher hazard of starting their ANC follow-up late than those who did experience bad obstetric outcomes, which is consistent with previous research [15]. Their perception of not being sick could have accounted for this. There is, therefore, the need to enlighten the women of the holistic benefits of early initiation of ANC.

The quantitative approach of this study also revealed that pregnant women with a history of known chronic illness were 57.1% less likely to initiate ANC visit early than their counterparts, which is not in line with any previous undertaking. Appreciating the fact that more than 12% of those pregnant women were patients leaving with HIV who did not want to disclose their status, for which they took, even, their medication from other distant ART sites; they may not early present themselves to the nearby health facilities, where they think their status will no longer be confidential [16].

The hazard of late ANC visit initiation for the pregnant women who were not accompanied by their partners during ANC visit for the current pregnancy was increased by 78.8% in contrast to those accompanied by their partners. This is supported by a study done in Kembata Tembaro Zone, Southern Ethiopia in 2014 [17]. This study also indicates that pregnant women who accessed ANC providing health facilities within a 5-kilometer radius have a 1.62 times higher probability of timely initiating their ANC visit than their contemporaries, which is in line with previous works [18, 19]. This might be women who live distant to the maternity facility may impose an extra cost for transportation service as well as lack of availability of transportation and therefore they fail to attain the health facility for receiving ANC services timely.

Being uncertain whether the pregnancy had occurred or not was found to be one of the major reasons why pregnant women in Legambo district, Ethiopia late initiate ANC visit in our qualitative analysis. A study from Easter Zone Tigray, Ethiopia support this finding of the current study by revealing that women who recognize their pregnancy earlier were more likely to early initiate the ANC services than those who recognize their pregnancy later [20]. In addition, the belief that one is at low risk of pregnancy-related complications was one of the major reasons that women initiated ANC visits late. A study from Cameroon and Axum, Ethiopia supports this finding [21, 22]. The participants in this study thought that the absence of abnormal symptoms during pregnancy is a reflection of the normality of the fetus inside, and they believe going early for an ANC visit is not necessary. This might be because of antenatal care is perceived by the mothers as curative rather than the preventive measure that is why the mothers start ANC late if they not get any pregnancy related illness.

Cultural beliefs, myths, and behavioral determinants of ANC initiation time was also found from the qualitatively exploration. Accordingly, the socio-cultural beliefs and myths called ‘Qare’ results in a late ANC initiation in the current study. Pregnant women in this community are advised not to expose themselves early at public gatherings. They believe that a pregnant woman can see another pregnant woman with a problem in the gathering, and this problem will happen to a healthy pregnant woman. Similarly, other studies identified the different types of cultural practices that make women not start ANC early [23–25]. For instance, in Malawi, there is a cultural practice called "Kuthimba", in which women wait for marriage counselors from their husband’s side to come and give them advice before starting ANC. Besides, women hide the pregnancy in the early months to avoid being bewitched [23]. This all makes women not start ANC early. This implies that it is crucial to understand and consider local beliefs and cultural practices when attempting to improve early ANC initiation.

### Strength and limitation of the study

The study’s strength is that it used a combination of qualitative and quantitative methods to gather reliable information and further investigate why women initiate ANC follow-up late. However, it will not be free from limitations. Using a medical chart review for the quantitative analysis by which we did not have full control over the choices of independent variables was the major limitation of this study. Being relied on the preexisting data that was unable to verify independently might made us to introduced recall bias. However, professionally measured outcomes would have neutralized the likely effect of such biases and the study tried, all the best possible, to include likely predictors considered by primary databased surveys. (i.e., distance measurement using the Global Positioning System) and supported by the qualitative study.

## Conclusions

The initiation time of the first ANC visit among women in Legambo district was relatively late. According to the findings of this study, pregnant women who were not supported or accompanied by their partners during her ANC visit, had unknown LNMP, had no or few live children, had no bad obstetric history, had a known chronic illness, were unsure about their pregnancy, and perceive they were not at risk; those who were influenced by social networks and socio-cultural beliefs and myths, had negative experience with healthcare providers, and lived within a radius of >5-kilometer from health facilities were most affected by late ANC initiation. Therefore, it is better to give great emphasis on the initiation time of ANC beyond simply having ANC visits and give attention to strengthening the pregnant women identification program and performing regular pregnant women’s conference to achieve early initiation of antenatal care visits. To lessen the negative influence of their immediate contacts, it will also good to include these old moms and husbands in the conference of pregnant women. Interventions to address ANC initiation need to accommodate local beliefs; home visits would be a potential alternative, or appointment systems that reduce joint meetings could be considered. Efforts should also be done to meet the world health organization recommendation for the accessibility of health facilities to pregnant women.

## Supporting information

S1 Data Dataset in Stata format. (DTA)

## Abbreviations

AHR: Adjusted Hazard Ratio
ANC: Antenatal Care
ART: Antiretroviral Therapy
CHR: Crude hazard Ratio
CI: Confidence Interval
FGD: Focused group Discussion
HDA: Health Development Army
HEW: Health Extension Workers
HIV: Human Immunodeficiency Virus
LICs: Low-income Countries
LNMP: Last Normal Menstrual Period
MMR: Maternal Mortality Ratio
SSACs: sub-Saharan Africa Countries
